# Reduced 5-Methylcytosine Level as a Potential Progression Predictor in Patients with T1 or Non-Invasive Urothelial Carcinoma

**DOI:** 10.3390/ijms16010677

**Published:** 2014-12-30

**Authors:** Chi-Jung Chung, Chao-Hsiang Chang, Chih-Pin Chuu, Chi-Rei Yang, Yi-Huei Chang, Chi-Ping Huang, Wen-Chi Chen, Mu-Chi Chung, Han Chang

**Affiliations:** 1 Department of Health Risk Management, College of Public Health, China Medical University, Taichung 40402, Taiwan; E-Mail: cjchung1010@gmail.com; 2 Department of Medical Research, China Medical University Hospital, Taichung 40402, Taiwan; 3 Department of Urology, China Medical University and Hospital, Taichung 40402, Taiwan; E-Mails: d8395@mail.cmuh.org.tw (C.-H.C.); cryang@mail.cmuh.org.tw (C.-R.Y.); D21959@mail.cmuh.org.tw (Y.-H.C.); d17561@mail.cmuh.org.tw (C.-P.H.); wgchen@mail.cmu.edu.tw (W.-C.C.); 4 Department of Medicine, College of Medicine, China Medical University and Hospital, Taichung 40402, Taiwan; 5 Institute of Cellular and System Medicine, National Health Research Institutes, Miaoli 35053, Taiwan; E-Mail: cpchuu@nhri.org.tw; 6 Translational Center for Glandular Malignancies, National Health Research Institutes, Miaoli 35053, Taiwan; 7 Graduate Program for Aging, China Medical University, Taichung 40402, Taiwan; 8 Division of Nephrology, Department of Internal Medicine, Taichung Veterans General Hospital, Taichung 40705, Taiwan; E-Mail: mcchung0322@gmail.com; 9 Department of Pathology, College of Medicine, China Medical University and Hospital, Taichung 40402, Taiwan

**Keywords:** 5-methylcytosine, immunohistochemistry, urothelial carcinoma, DNA (cytosine-5)-methyltransferase 1

## Abstract

This study aims to elucidate the level of DNA methylation in urothelial carcinomas (UCs) using 5-methylcytosine (5-MeC) immunohistochemistry (IHC). We examined the relationship among 5-MeC levels, DNA (cytosine-5)-methyltransferase 1 (DNMT1) immunostaining levels, and clinicopathologic features. Tissue samples included 23 normal urothelia and 150 urothelial neoplasia, which comprised 40 non-invasive and 110 invasive UCs. The levels of 5-MeC and DNMT1 were assessed based on their immunoreactivities and then divided into low and high levels. In addition, we collected information on clinical variables, pathologic features, and recurrent status from patient questionnaires and medical records. Chi-square test and multivariate logistic regression model were used for analyses. Results showed that 5-MeC levels were positively associated with DNMT1 levels in UC (*p* = 0.0288). Both 5-MeC and DNMT1 were low in approximately 50% (76/150) of UC. The percentage of low 5-MeC levels was higher in invasive UC (65/110; 59%) than in normal urothelia (2/23; 13%) and non-invasive UC (18/40; 45%). Clinical factors were independently associated with low 5-MeC levels after adjusting for age and sex, including cancer stages II–IV, presence of UC *in situ*, and marked inflammation. Low 5-MeC levels in stage I invasive UC were not significantly different from those of non-invasive tumors (*p* = 0.8478). Low DNMT1 levels were only associated with UC with squamous differentiation (*p* = 0.0365). Neither 5-MeC nor DNMT1 levels were associated with UC recurrence. In conclusion, a low 5-MeC level could predict the progression of UC invasion into muscle.

## 1. Introduction

Urothelial carcinomas (UCs) are exclusively derived from the urothelium throughout the urinary tract, including the renal pelvis, ureter, urinary bladder, and urethra. The urinary bladder is the most common site, with the main environmental risk factors being cigarette smoking, occupational exposure to aromatic amines, chlorinated hydrocarbons, and inorganic arsenic from drinking water [1]. The possible mechanisms of carcinogenesis, such as chromosomal aberrations, aberrant DNA methylation, and loss of function of p53 or other tumor suppression genes, have been previously investigated [1].

DNA methylation is a natural modification that requires the addition of a methyl group to the 5' position of the cytosine ring in the context of CpG dinucleotides to form 5-methylcytosine (5-MeC). Regional DNA hypermethylation and global DNA hypomethylation have been reportedly linked with human carcinogenesis [2,3,4,5]. Regional DNA hypermethylation in the promoter region and decreased expression of tumor suppressor genes, such as *p16* and *E-cadherin*, have been reported in UC [6,7]. Increased levels of genome-wide DNA methylation in relation to poor prognosis have been explored in UC [4]. Additionally, DNA (cytosine-5) methyltransferase 1 (DNMT1) is an enzyme responsible for maintaining methylation patterns [5]. The correlation of increased DNMT1 mRNA and protein expression with increased DNA methylation on CpG islands has been reported in Epstein–Barr virus-associated gastric cancers and UC [8,9].

DNA hypomethylation could result in genetic instability because of alterations in the chromatin structure; this instability could be considered as an early biomarker of human carcinogenesis [10,11,12,13]. Compared with corresponding non-tumor tissues, DNA hypomethylation has been detected in precancerous conditions and cancers of the colorectum, stomach, and prostate, suggesting that global DNA hypomethylation may occur at the precancerous stage [10,12,13]. A large case-control study has revealed that DNA hypomethylation is associated with increased risk for developing bladder cancer [14]. DNA hypomethylation could also be a prognostic factor when patients have pT1a renal cell carcinomas [15]. However, the relationship among DNA methylation levels, DNMT1 level, and clinicopathologic features of UC has not been reported to date.

Global DNA methylation assays, which are used to detect the global DNA methylation level in tissue samples or peripheral blood, include capillary electrophoresis and mass spectrometry, gas chromatography–mass spectrometry, methyl group acceptance assay, immunohistochemistry (IHC), and *Cp*Global assay [16,17,18,19]. Among the assays, 5-MeC IHC is the most convenient and quickest test for tissue samples. In this study, we examined the level of DNA methylation in UC using 5-MeC IHC. We also examined the association between 5-MeC levels and DNMT1 immunostaining levels, as well as that between 5-MeC levels and clinicopathologic features. Data generated from this study provide evidence that low 5-MeC could predict the progression of UC invasion into the muscle.

## 2. Results and Discussion

### 2.1. Study Subjects

The 150 UC samples included 65 males and 85 females. Their ages ranged from 26 to 87 years with a median of 68 years (Table 1). Most UCs were invasive (including 108 invasive UCs and two small cell carcinomas), high grade, and located at the upper urinary tract. Stage 0 UC accounted for 26.6%, stage I for 28.0%, and stages II–IV for 45.4% of the total samples. Moreover, 40.0% of the UC samples showed subjacent UC *in situ* (UIS). Subjacent UIS was described as a status of invasive UC concomitant with UIS. Approximately 14.7% (22/150) of UC samples showed marked inflammation. Inflammation was defined as the inflammatory background within the tumors, and its severity was quantified by the increasing amounts of lymphoplasmacytic infiltrates: none, + (mild), ++ (moderate), and +++ (severe with lymphoid aggregates). Other detailed clinical features are presented in Table 1.

**Table 1:** Clinicopathologic variables of patients with urothelial carcinomas (UCs).

Variables		*n* (%)	*H*-Score (Mean ± SD)	
			5-MeC	DNMT1
All cases		150 (100.0)	104.9 ± 23.2	162.3 ± 19.3
Age (year)	Median (range)	68 (26–87)	–	–
Sex	Male	65 (43.3)	109.9 ± 25.6	163.5 ± 19.9
	Female	85 (56.7)	101.0 ± 20.5	161.4 ± 8.9
Smoking	Never	66 (44.0)	108.5 ± 24.1	161.8 ± 17.3
	Ever	36 (24.0)	109.4 ± 22.5	159.9 ± 23.3
	Missing cases	48 (32.0)	–	–
Tumor histology	UIS	5 (3.3)	126.8 ± 18.3	174.8 ± 21.3
	Noninvasive papillary UC	35 (23.3)	110.7 ± 24.2	162.4 ± 22.7
	Invasive UC	108 (72.0)	102.1 ± 23.3	161.7 ± 18.1
	Small cell carcinoma	2 (1.4)	115.7 ± 3.9	149.6 ± 0.38
Tumor location	Pelvis	27 (18.0)	108.7 ± 22.4	163.2 ± 17.6
	Ureter	65 (43.3)	98.0 ± 20.8	161.4 ± 17.4
	Urinary bladder	58 (38.7)	110.9 ± 24.3	162.8 ± 22.2
Tumor grading	Low grade	34 (22.7)	107.3 ± 25.1	166.1 ± 20.7
	High grade	116 (77.3)	104.2 ± 22.7	161.2 ± 8.9
TNM stage	0a	35 (23.3)	110.7 ± 4.2	162.4 ± 22.7
	0is	5 (3.3)	126.8 ± 18.3	174.8 ± 1.3
	I	42 (28.0)	105.0 ± 22.9	162.9 ± 18.6
	II	34 (22.7)	100.3 ± 20.2	159.2 ± 19.0
	III	33 (22.0)	99.8 ± 24.2	162.1 ± 6.5
	IV	1 (0.7)	113.2	183.5
Tumor recurrence	Absent	134 (89.3)	105.2 ± 23.6	162.9 ± 18.7
	Present	16 (10.7)	102.3 ± 20.1	157.5 ± 24.3
*Pathologic Features*				
Subjacent UIS	Absent	90 (60.0)	109.3 ± 22.3	164.2 ± 19.2
	Present	60 (40.0)	98.4 ± 23.1	159.4 ± 9.3
Inflammation	None	54 (36.0)	107.4 ± 24.8	163.7 ± 19.4
	+	41 (27.3)	108.4 ± 19.8	167.4 ± 19.1
	++	33 (22.0)	103.5 ± 25.2	160.6 ± 15.9
	+++	22 (14.7)	94.5 ± 19.6	151.9 ± 21.2
Tumor with SD	Absent	107 (71.3)	106.5 ± 22.6	163.5 ± 19.8
	Present	43 (28.7)	100.9 ± 24.5	159.3 ± 18.1
Tumor with GD	Absent	137 (91.3)	105.3 ± 23.7	162.9 ± 19.3
	Present	13 (8.7)	100.4 ± 17.4	155.3 ± 19.2
LVI or PNI	Absent	128 (85.3)	105.2 ± 23.2	161.6 ± 19.7
	Present	22 (14.7)	103.2 ± 23.7	166.3 ± 17.3

*H*-Score, representative of the staining intensity and described in “Experimental Section”; 5-MeC, 5-methylocytosine; DNMT1, DNA (cytosine-5)-methyltransferase 1; 0a, noninvasive papillary carcinoma; 0is, carcinoma *in situ*; UIS, urothelial carcinoma *in situ*; UC, urothelial carcinoma; Subjacent UC *in situ* is described as a status of invasive UC concomitant with UC *in situ*; Inflammation is defined as tumor stromal inflammation, and its severity is quantified as none, + (mild), ++ (moderate), and +++ (severe with lymphoid aggregates); Tumor with squamous (SD) or glandular differentiation (GD) is defined as a case of more than 10% of cancer cells having squamous or glandular differentiation; Lymphovascular invasion (LVI) is defined as cancer emboli present in lymphovascular channels; Perineural invasion (PNI) is defined as cancer cells invading nerve bundles.

### 2.2. Correlation of 5-MeC and DNMT1 Levels

**Figure 1:**
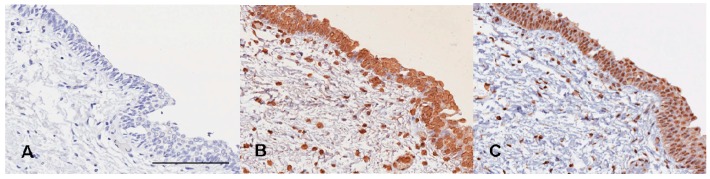
Normal urotheliun. (**A**) Mouse IgG control; (**B**) 5-Methylocytosine (5-MeC) immunohistochemistry; (**C**) DNA (cytosine-5)-methyltransferase 1 (DNMT1) immunohistochemistry. High levels of both 5-MeC and DNMT1 present in the normal urothelium. Bar = 200 µm.

**Figure 2:**
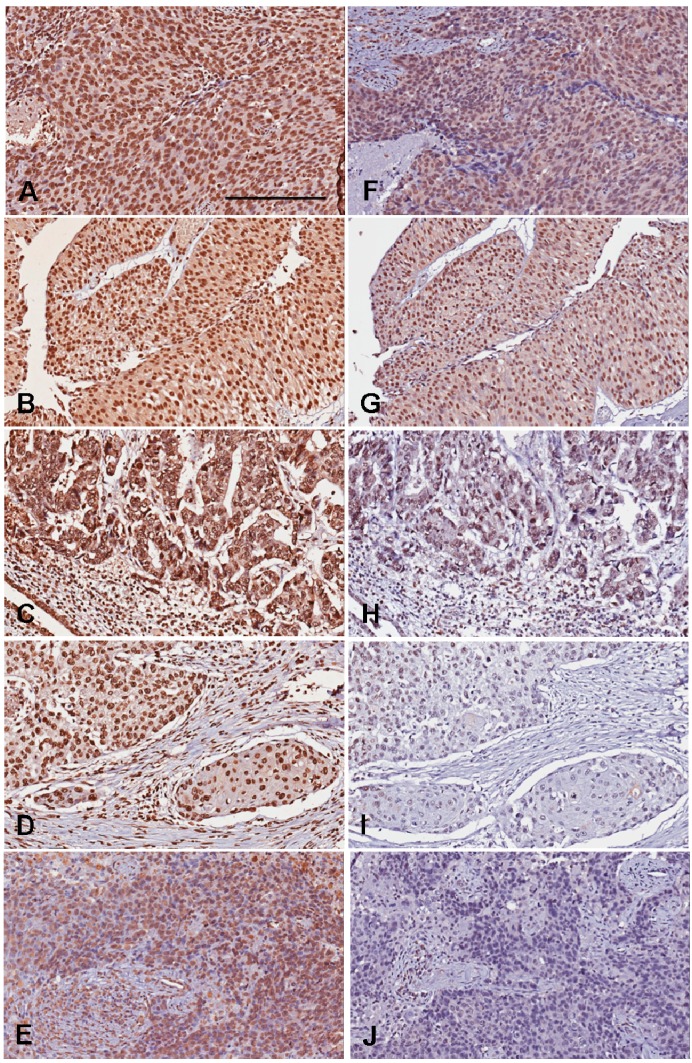
Representative photography of 5-MeC and DNMT1 immunohistochemistry. The 5-MeC photography in **A**–**E**; The DNMT1 photos in **F**–**J**. (**A**,**F**) High-grade urothelial carcinoma showing high levels of both 5-MeC and DNMT1; (**B**,**G**) Low-grade urothelial carcinoma showing high levels of both 5-MeC and DNMT1; (**C**,**H**) High-grade urothelial carcinoma with glandular differentiation, showing high 5-MeC but low DNMT1 levels. (**D**,**I**) High-grade urothelial carcinoma with squamous differentiation, showing low levels of both 5-MeC and DNMT1; (**E**,**J**) High-grade urothelial carcinoma showing low levels of both 5-MeC and DNMT1. Bar = 200 µm.

Levels of 5-MeC and DNMT1 for UC are shown in Table 1. The mean basal levels (H-scores) of 5-MeC and DNMT1 in normal urothelia were used as cut-off values (Figure 1); then, a total of UC samples were categorized into low and high levels of 5-MeC or DNMT1 according to H-scores (Figure 2).

The percentage of low 5-MeC level in normal urothelia was 13.0% (2/23), upper urinary tract 63% (58/92) and urinary bladder 43% (25/58) (Table 2). All normal urothelia showed high DNMT1 levels. Out of 150 patients with UC, the percentage of low 5-MeC accounted for 55.4% (83/150), whereas that of low DNMT1 comprised 86.0% (129/150). A positive correlation was found between 5-MeC and DNMT1 levels with statistical significance (*p* = 0.0288, chi-square test). Approximately 50.7% (76/150) of UC patients presented low 5-MeC and DNMT1 levels.

**Table 2:** Distribution of 5-MeC levels in different tumor locations and correlations of 5-MeC and DNMT1 levels for UCs using immunohistochemistry (*n* = 150).

Variables		5-MeC Level		
		Low	High	*p*-Value
Tumor location	Normal urothelia (*n* = 23)	2	21	
	Pelvis (*n* = 27)	15	12	0.0014 *
	Ureter (*n* = 65)	43	22	<0.0001 *
	Urinary bladder (*n* = 58)	25	33	0.0069 *
*For 150 UC Tissue Samples*				
DNMT1 level	Low (*n* = 129)	76 (50.7%)	53 (35.3%)	0.0288 ^†^
	High (*n* = 21)	7 (4.7%)	14 (9.3%)	

5-MeC, 5-methylocytosine; DNMT1, DNA (cytosine-5)-methyltransferase 1. Percentage in bracket indicates positive number/total number. * Significant when *p*-value <0.05 using chi-square test compared to normal urothelia; ^†^ Significant when *p*-value <0.05 using Chi-square test.

### 2.3. Relationship among 5-Methylocytosine (5-MeC) Level, Clinicopathologic Features, and Urothelial Carcinomas (UC) Recurrence

Statistical analyses in Table 3 and Table 4 demonstrate that females, cancer stages II–IV, ureteral UC, subjacent UIS, and marked inflammation were independently associated with low 5-MeC level. No association of low 5-MeC level was found with age, smoking history, tumor grade, and tumor recurrence. Stage I invasive UC was not significantly different from non-invasive tumors (*p* = 0.8478, multivariate logistic regression, Table 4). The pathological features, including squamous differentiation (SD), glandular differentiation (GD), lymphovascular invasion (LVI), and perineural invasion (PNI), were also not associated with low 5-MeC level. SD or GD was defined as the case in which more than 10% of UC cells presented SD or GD. LVI and PNI were used to describe the case in which cancer emboli were present in lymphovascular channels and cancer cells invaded nerve bundles, respectively. For DNMT1 levels, a low level was only associated with UC with SD (*p* = 0.0365, chi-square test, Table 3). No other independent associations were observed between DNMT1 level and either clinical or pathological features (Table 4).

**Table 3:** Comparison of UCs with low 5-MeC or DNMT1 level stratified by clinicopathological features (*n* = 150).

Variables		All Cases	Biomarkers with Low Level, *n* (%)			
			5-MeC	*p*-Value	DNMT1	*p*-Value
Age	<68	73	43 (58.9)	0.3917	64 (87.7)	0.5657
	≥68	77	40 (52.0)		65 (84.4)	
Sex	Male	65	30 (46.2)	0.0480 *	54 (83.1)	0.3669
	Female	85	53 (62.4)		75 (88.2)	
Smoking †	Never	66	35 (53.0)	0.4072	55 (83.3)	0.7124
	Ever	36	16 (44.4)		31 (86.1)	
Tumor location	Pelvis	27	15 (55.6)	0.0371 *	22 (81.5)	0.1500
	Ureter	65	43 (66.2)		60 (92.3)	
	Urinary bladder	58	25 (43.1)		47 (81.0)	
Tumor grade	Low grade	34	18 (52.9)	0.7500	30 (88.2)	0.6690
	High grade	116	65 (56.0)		99 (85.3)	
TNM stage	0a/0is	40	18 (45.0)	0.0321 *	33 (82.5)	0.7250
	I	42	20 (47.6)		36 (85.7)	
	II/III/IV	68	45 (66.2)		60 (88.2)	
Tumor recurrence	Absent	134	75 (56.0)	0.6493	115 (85.8)	0.8548
	Present	16	8 (50.0)		14 (87.5)	
*Pathologic Features*						
UIS	Absent	90	41 (45.6)	0.0032 *	74 (82.2)	0.1024
	Present	60	42 (70.0)		55 (91.7)	
Inflammation	None/+/++	128	65 (50.8)	0.0068 *	109 (85.2)	0.7402
	+++	22	18 (81.8)		20 (90.9)	
SD	Absent	107	56 (52.3)	0.2442	88 (82.2)	0.0365 *
	Present	43	27 (62.8)		41 (95.4)	
GD	Absent	137	74 (54.0)	0.2916	117 (85.4)	0.6948
	Present	13	9 (69.2)		12 (92.3)	
LVI or PNI	Absent	128	71 (55.5)	0.9359	110 (85.9)	0.9576
	Present	22	12 (54.6)		19 (86.4)	

* Significant when *p*-value <0.05 using chi-square test; † 48 missing cases.

**Table 4:** Significant factors associated with UCs with low 5-MeC or low DNMT1 level.

Independent Variables	Dependent Variable: 5-MeC				Dependent Variable: DNMT1	
	OR (95% CI)		*p*-Value		OR (95% CI)	*p*-Value
Age (≥68 compared with <68)	0.74 (0.39–1.43)		0.3752		0.76 (0.30–1.93)	0.5594
Sex (Female compared with Male)	1.95 (1.01–3.76)		0.0476 *		1.53 (0.61–3.87)	0.3656
Smoking (Ever compared with Never)	1.32 (0.41–4.26)		0.6416		2.98 (0.67–13.15)	0.1499
Tumor recurrence (Present compared with Absent)	0.89 (0.31–2.58)		0.8336		1.28 (0.27–6.21)	0.7570
Tumor Location						
Pelvis compared with Urinary bladder	1.46 (0.57–3.77)		0.4302		2.82 (0.90–8.88)	0.0762
Ureter compared with Urinary bladder	2.55 (1.20–5.44)		0.0150 *		0.95 (0.28–3.16)	0.9264
Tumor grade (High compared with Low)	0.86 (0.42–1.76)		0.6779		0.71 (0.25–2.02)	0.5206
TNM Stage						
I compared with 0a/0is		1.09 (0.45–2.63)		0.8478	1.33 (0.40–4.40)	0.6365
II–IV compared with 0a/0is		2.76 (1.20–6.35)		0.0168 *	1.65 (0.54–5.03)	0.3806
UIS (present compared with absent)		2.76 (1.37–5.56)		0.0045 *	2.34 (0.80–6.79)	0.1190
Inflammation (+++ compared with None/+/++)		4.96 (1.53–16.08)		0.0076 *	1.85 (0.39–8.81)	0.4379
SD (present compared with absent)		1.38 (0.66–2.91)		0.3917	4.23 (0.93–19.20)	0.0620
GD (present compared with absent)		2.09 (0.59–7.35)		0.2519	2.21 (0.27–18.31)	0.4630
LVI or PNI (present compared with absent)		0.94 (0.37–2.36)		0.8925	1.01 (0.27–3.79)	0.9851

OR, odds ratio; CI, confidence interval. * Significant when *p*-value < 0.05 using multivariate logistic regression after adjusting for age and sex.

Sixteen patients with UC showed local or distant recurrences of tumor. The mean recurrence-free interval was 3044 ± 107 day for patients with low 5-MeC cancers and 3100 ± 163 day for those with high 5-MeC cancers (*p* = 0.610, log-rank test). The mean recurrence-free interval was 3171 ± 103 day for patients with low DNMT1 cancers and 2467 ± 162 day for those with high DNMT1 cancers (*p* = 0.879, log-rank test). Therefore, the levels of 5-MeC or DNMT1 did not affect recurrence-free survival for patients with UC in this cohort.

### 2.4. Discussion

This study was the first to evaluate the relationships among 5-MeC levels, DNMT1 levels, and clinicopathologic features using IHC in UC samples. Results showed that low 5-MeC samples were more common in invasive UC than in normal urothelia and non-invasive UC. Low 5-MeC levels were significantly associated with cancer stages II–IV, presence of UIS, and UC with severe inflammation. The 5-MeC levels of stage I invasive UC were not significantly different from those of non-invasive tumors. These findings may imply that low 5-MeC could predict the progression of UC invasion into muscle.

Genetic studies linked to tumor histology, grading, and invasiveness of UC were separated into two genomic subgroups: chromosomally stable tumors associated with fibroblast growth factor receptor 3 (*FGFR3*) mutations and non-invasive UC and chromosomally unstable tumors associated with *p53* mutations, chromosome 9 abnormalities, and muscle-invasive UC [20]. No evidence proves that a link exists between global DNA hypomethylation and p53 or FGFR3 epigenetics in UC. Although the precise reason for DNA hypomethylation remains unknown, the loss of DNMTs is considered an important factor [21]. Animal models of DNMT1 knockout mice have demonstrated that the lack of DNMT1 activity is associated with decreased genomic methylation level and increased frequency of cancer formation through chromosomal abnormality and instability [5]. Some *in vitro* assays that were consistent with the results of animal studies showed lower expressions of DNMT1 mRNA and protein in UC cells than in normal cell lines [22], as well as a strong correlation of chromosome instability, such as loss of heterozygosity of chromosome 9 [23]. DNMT3b has been also found to be involved in DNA hypomethylation in the pericentromeric satellite region [4]. In addition, inflammation could affect DNMT expression [4,24,25]. In inflammation-associated carcinogenesis, hepatocellular carcinoma, pancreatic cancer, and virus-related gastric cancer all reveal a positive association between DNMT1 expression and level of regional DNA methylation [4]. Moreover, the different cytokines inducing variable expressions of DNMT1 have been studied in different inflammatory diseases [24,25]. For example, proinflammatory cytokine IL-1 decreased DNMT1 mRNA and protein expression in rheumatoid arthritis synoviocytes, suggesting that chronic IL-1 exposure could be a DNMT inhibitor. By contrast, IL-6 increased DNMT1 expression involving gene silencing in colon cancer cells. These cytokine studies could provide a possible explanation for the finding that DNMT1 level is unrelated to inflammation status in UC samples.

In addition to loss of DNMTs, several factors are believed to be involved in a low-5-MeC process. Oxidative stress is linked to the production of free radical adducts that induce alterations of DNA methylation level and DNA integrity [26]. Folate functions in one-carbon metabolisms influence methyl groups and the methylation process [2,27]. A study revealed that folate deficiency is associated with oxidative DNA damage and global DNA hypomethylation in workers chronically exposed to chromate [28]. A similar finding on human colon cells showed that folate depletion induces global DNA hypomethylation, including that in the *p53* region. Taken together, types of DNMT, status of disease inflammation, actions of cytokines and environmental or nutritious effects might involve, at least partially, the DNA methylation directly or indirectly.

Long interspersed nuclear element-1 (*LINE-1*) gene comprises 17% of the total human genome and is one of the most studied sequences for DNA methylation [29]. Therefore, the methylation level of the *LINE-1* gene is a proxy of global DNA methylation. Van Bemmel *et al.* investigated global methylation levels in UC samples by detecting the *LINE-1* gene and found decreasing methylation levels in the order of non-tumor urothelia, non-invasive UC, and invasive UC [30]. Seifert *et al.* used IHC in exfoliative urothelial cells in the urine of UC patients and found a lower DNA methylation level compared with that in healthy individuals [31]. Their results are comparable to our findings using the IHC in UC tissues. To facilitate a comparison with our experiment, we assessed the DNA methylation level by using imaging software to account for the *H*-scores and to avoid bias from inter-individuals and subjective interpretation. We also used phosphate and IgG controls to examine the specificity of the primary and secondary antibodies. Although the DNA methylation levels evaluated by the IHC method could be relative and not actual levels of DNA methylation, Hernandez–Blazquez *et al.* investigated the IHC pattern and intensity of 5-MeC in colonic cancers and found that the IHC method is feasible and could provide information on levels of DNA methylation [32].

Some limitations of this study have to be discussed. The normal urothelium microarray was commercial, and the sample size was too small to increase statistical power. The mean basal level of 5-MeC was obtained from normal urothelia. The human tissues used in the IHC were obtained from individuals who underwent invasive procedures; thus, IHC is difficult to apply to a general population.

## 3. Experimental Section

### 3.1. Ethics Statement

One normal urothelium microarray including 23 tissue cores and four UC microarrays comprising 150 tissue cores were used. The normal urothelium microarray was purchased from US Biomax, Inc. (Rockville, MD, USA). The UC samples on the tissue microarrays were collected from the Department of Pathology, China Medical University Hospital (CMUH), between 2005 and 2012. All tumor histologies and grades were confirmed by a pathologist. This study was approved by the Institutional Review Board at CMUH, Taiwan (DMR100-IRB-262).

### 3.2. Clinical and Pathological Features

Clinical features included age, gender, smoking history, tumor location, tumor type, tumor grade, cancer stage, date of tumor recurrence, and pathological features, such as subjacent UIS, inflammation, tumor with SD, tumor with GD, and tumor with LVI or PNI. Data were acquired from medical records or pathology reports of patients.

Smoking history was recorded as “never” or “ever”. “Never” smoking history referred to subjects who have never smoked. Individuals who currently smoke or had smoked were designated as “ever”. The tumor locations comprised the renal pelvis, ureter, and urinary bladder. The tumor type and grade were diagnosed according to the criteria set by the World Health Organization. The UC samples were staged into 0a, 0is, and I to IV according to the tumor node metastasis criteria outlined in the American Joint Committee on Cancer Staging Manual. Stage 0a was pTaN0M0, indicating the non-invasive papillary UC. Stage 0is was pTisN0M0, indicating the UIS. Tumor recurrence was a local recurrence either *in situ* or in the urinary bladder or upper urinary tract, with distant metastasis. The recurrence-free duration was defined as the interval between the date of initial tumor diagnosis and the date of first recurrence as confirmed by pathological examination.

### 3.3. 5-MeC and DNA (Cytosine-5)-Methyltransferase 1 (DNMT1) Immunohistochemistry

Tissue microarray sections (4 μm thick) were dewaxed with xylene, rehydrated with serially decreased concentrations of alcohol, and bathed in a phosphate buffer. Primary specific antibodies included 5-MeC (1:500 dilution; Calbiochem, Darmstadt, Germany) and DNMT1 (1:200 dilution; Calbiochem, Darmstadt, Germany). Secondary antibody conjugated to a peroxidase-labeled polymer was purchased from Thermo Fisher (UltraVision Quanto Detection System; Thermo Fisher Scientific, Cheshire, UK). The sections were counterstained with hematoxylin. Mouse serum and phosphate buffer, rather than primary antibodies, were used as negative controls. Positive controls were normal urothelia for 5-MeC and DNMT1 IHC (Figure 1).

### 3.4. Assessments for 5-MeC and DNMT1 Immunoreactivities

**Figure 3:**
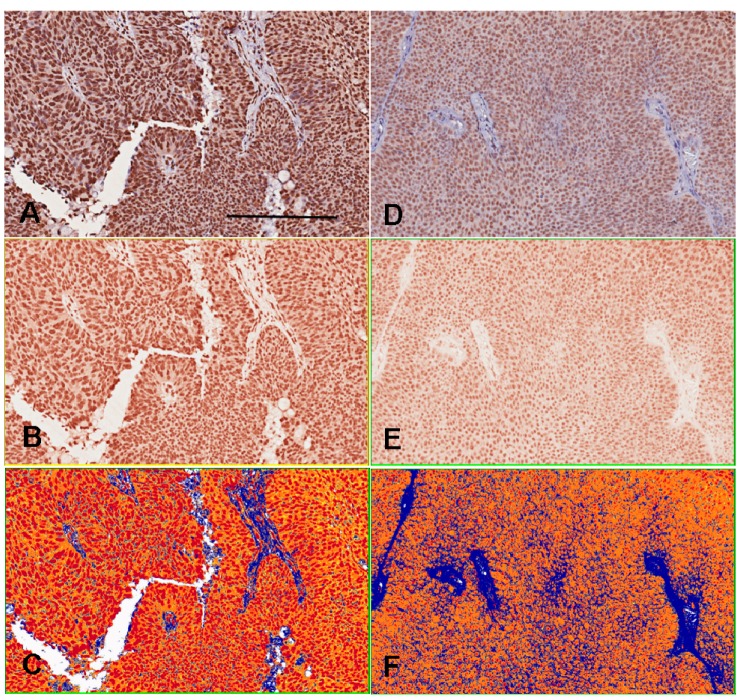
Immunohistochemical intensity representated as H-score by imaging software counts. The **A**–**C** photography are the sample 1. The **D**–**F** photography are the sample 2. The positive color in our experiment is brown. The photography (**A**,**D**) are initially changed to brown-color channels (**B**,**E**) before intensity counting. The positive pixel counts (**C**,**F**) shows red color as strong positivity (staining intensity grade 3), orange color as medium positivity (grade 2), yellow color as weak positivity (grade 1) and blue color as negativity (grade 0), and finally quantifies the areas and intensities to get *H*-scores ranged from 0 to 300. The H-score of sample 1 by 175 is higher than sample 2 by 133. Bar = 200 µm.

The 5-MeC- or DNMT1-positive cells presented a brown color and were localized at the nuclei and/or cytoplasm of the normal urothelia and urothelial cancer cells. The interstitial stroma exhibited 5-MeC and DNMT1 immunonegativity. H-scoring system was used to assess staining intensity by employing imaging software (Aperio Technologies Inc., Vista, CA, USA) to obtain an objective evaluation and avoid subjective interpretation. The grades of staining intensity were four-tiered as follows: 0, negative staining; 1, weak staining; 2, medium staining; and 3, strong staining. The *H*-score represented the sum of the mean value in each grade multiplied by the proportion of positive cells for each tissue core (Figure 3).

The normal urothelia showed the basal levels of 5-MeC and DNMT1. The mean basal levels (*H*-scores) of 5-MeC and DNMT1 in normal urothelial samples were 135.6 ± 30.8 and 183.3 ± 1.8, respectively. The cut-off values of 106 for 5-MeC level and 181 for DNMT1 level were considered as the 50th percentiles of the basal levels to distinguish high and low levels, respectively. UC values greater than 106 or 181 of the H-score were considered high 5-MeC or DNMT1 cancers; conversely, UC values less than or equal to 106 or 181 were considered low 5-MeC or DNMT1 cancers (Figure 2).

### 3.5. Statistical Analysis

Descriptive statistics was used to calculate the mean, standard deviation, frequency of all clinical and pathological features, and *H*-scores. Associations between category variables and expression patterns of 5-MeC and DNMT1 (high/low) were examined using the chi-square test. The odds ratios and 95% confidence intervals of low 5-MeC or low DNMT1 levels for UC were estimated using multivariate logistic regression models after adjusting for age and sex. Kaplan–Meier estimates and log-rank test were used to determine the recurrence-free survival time and to compare the differences in survival between low and high 5-MeC and DNMT1 levels. Two-sided *p* value less than 0.05 was considered significant. The analyses were conducted using SAS statistical package (SAS, version 8.0, Cary, NC, USA).

## 4. Conclusions

Low 5-MeC levels were identified in stages II–IV UC rather than in non-invasive or stage I UC. Thus, a low 5-MeC level could predict the progression of tumor invasion into muscle. 5-MeC IHC is an alternative method for determining DNA methylation levels in tissue samples.
